# Functions of the Nervous System

**Published:** 1857-12

**Authors:** 


### FUNCTIONS OF THE NERVOUS SYSTEM.

### THE NERVE CENTERS.

In my two former articles I endeavored to present an outline view of the forms and relations of the various animal nerve centers, and to arrange, in a way that would be available for the use of the junior students, the different animal nerves into groups, so that they may be understood in their various relations at a glance. It has already appeared that there are three distinct nerve centers of animal life, in addition to certain ganglia, which are the centers of the various nerves of special sense. Although these centers are entirely distinct, and, in a certain sense, independent in their functions, still they are, in various ways, combined or related in their operations, so as to greatly modify the use of the others.

Thus, the center of sensation and of motion exists in the lowest vertebrated animals; but in such beings, they only serve the purpose of imparting the faculty of sensation to pain from inflicted injury, and an indefinite power of motion, which is commonly exercised for the purpose of removing to a place where the injury can not be repeated. This function, which has been secured by these simple instruments, is called the reflex, or excito-motor function.

As before mentioned, in the lower vertebrates, this is the only animal nerve center; and, consequently, the source of the only proper animal nerve function. A being which is no more perfectly endowed with a nervous system is indeed very low in the animal scale. As we ascend the scale toward the most perfect animals, we find other apparatus being added, which gradually endow the being with a higher function. The apparatus that exalt the animal to a higher position, are situated within the cranium,—the form and general divisions of which were referred to in a former number. They are the cerebrum and the cerebellum. These organs, although in immediate relation, differ in their intimate structure, and entirely diverse in their functions.

The spinal cord may be regarded both as the center of reflection, or of reflex action, and also the medium of voluntary motion. Two electrical currents (so to speak) pass along the same conductors, and can act simultaneously or separately, to suit the purposes of the operator.

The cerebrum, then, is the center of intelligence, and, as such, imparts to the spinal cord the ability to act under the controlling impulse of an intelligent will. A being endowed with this highest nerve center, which presides over the highest animal nerve function, would be degraded to a condition low indeed, without a spinal cord and its nerves, through the agency of which its mandates are obeyed. The cerebrum, then, is the center of thought, of intelligence, and of voluntary action, while it remains passive itself, so far as change of its own relation is concerned. It is the supreme dictator of animal motions, inasmuch as all voluntary action is performed under the immediate influence of its dictation.

Although it is the centre of all intelligent emotion, and is justly regarded as the highest nerve centre; for it exists only in the higher animals and is found in the greatest perfection in men, it, like the other centres, is not susceptible of a knowledge of injury to itself. It does not suffer; the animal does not suffer when the greatest violence is inflicted upon it.

The cerebellum, or the lesser train, which is but one seventh the size of this, and is situated in immediate relation with it, or beneath the posterior lobes of the cerebrum, also performs its own peculiar and independent function. It seems to be the office of this centre, to control, and to combine muscular action, so that the various muscles which are destined to unite in executing a given purpose, may act in harmony.

It is readily observed that there is very great diversity in degree of muscular activity, as well as of power to control rapid muscular motions, in different classes of animals. It is also observed that those animals which can exercise these faculties in greatest perfection, are endowed with a proportionally larger cerebellum. For example, the swallow is known to possess great muscular power, while it has the ability to control its motions with wonderful precision. This bird is found to possess a proportionally larger cerebellum; and it is in accordance with this principle that such birds as are inferior in this respect, have a proportionally smaller cerebellum. It has also been ascertained that the cerebellum may be laid bare, without impairing muscular harmony of action; also, that the organ may be sliced away by degrees, without the infliction of pain, while every slice that is removed gradually impairs the power of combination of muscular motion. When the organ is removed down to the crura, the animal can still see a blow that may be aimed at it, which it may vainly strive to avoid; but having lost the power of combination of muscular action, such efforts to escape are as likely to be made toward the source of injury as in the opposite direction. It will be perceived, then, that the cerebrum may direct, and the spinal cord may perform its functions with undiminished force, while the being is rendered helpless by the absence of ability for using its organs in harmony.

